# Scaling up social entrepreneurship to reduce poverty: Exploring the challenges and opportunities through stakeholder engagement

**DOI:** 10.3389/fsoc.2023.1131762

**Published:** 2023-03-13

**Authors:** Hari Harjanto Setiawan, Tauchid Komara Yuda, Badrun Susantyo, Muhammad Belanawane Sulubere, Mery Ganti, Habibullah Habibullah, Muslim Sabarisman, Ruaida Murni

**Affiliations:** ^1^National Research and Innovation Agency (BRIN), Jakarta, Indonesia; ^2^Social Development and Welfare, Universitas Gadjah Mada, Yogyakarta, Indonesia; ^3^Sociology and Social Policy, Lingnan University, Hong Kong, China

**Keywords:** poverty, empowerment, social entrepreneurship, stakeholder engagement, relationship

## Introduction

Stakeholder engagement is critical in state-driven social entrepreneurship programs. This engagement is based on the principle of mutual benefit in partnering through the parties' contributions following their respective roles and capacities. This paper explores stakeholder engagement in social entrepreneurship programs in Indonesia. This program is a program of the Indonesian government to reduce poverty. The issue of poverty is the primary concern of the Indonesian government and the international world. The COVID-19 pandemic has impacted increasing poverty because many people have lost their jobs due to policies limiting activities (Laborde et al., 2021). In addition to the ongoing social protection program, the Indonesian government is also working to reduce poverty through the Social Entrepreneurship Program.

Social Entrepreneurship is one of the essential factors in sustainable economic development (Lateh et al., 2018). Social entrepreneurship is different from entrepreneurship because it has a social mission and is not only concerned with profit. This program improves the poor's economic welfare by combining business and social dimensions. Research in the South Punjab region of Pakistan shows that empowerment through social entrepreneurship can significantly reduce poverty (Abrar ul haq et al., 2019).

Some parts of Tehran, Iran, implement empowerment strategies through social entrepreneurship to lift marginalized people from poverty (Sadabadi and Rahimi Rad, 2021). Social entrepreneurship can contribute to 10% of the gross domestic product in Kenya (Ngare, 2021). South Korea and Malaysia can increase regional economic growth by creating jobs (Doh, 2020; Mustaffa et al., 2020). Thus, this literature argues that poverty can be overcome through social entrepreneurship. Social entrepreneurship leads to increased innovation, employment opportunities, and access to capital. In the end, social entrepreneurship will succeed with sustainable development.

The Indonesian government implements social entrepreneurship programs in collaboration with non-state actors. This is what distinguishes between developing countries and developed countries. Most entrepreneurship in developed countries is supported by large companies that do not depend on state funding. This paper explores the involvement of stakeholders from two groups, namely state, and non-state. Our research also examines the relationship between state and non-state actors in negotiating program implementation. We also look at the impact of involving different stakeholders on the beneficiary communities.

This paper will answer three main hypotheses: first, each stakeholder group's role is expected to be a complementary relationship. Second, programs offered by the state will be accepted by non-state actors. Third, programs that involve many stakeholders will have a good impact on beneficiaries.

## Theoretical framework

### Poverty

Poverty is a condition where the basic needs of a decent life are not fulfilled, and the facilities and infrastructure are inadequate (Govender et al., 2007). Bradshaw (2009) defines poverty as a condition where basic food, shelter, health, and safety needs are unmet based on human rights values. When viewed from an economic aspect, poverty refers to the gap between weak purchasing power and the desire to meet basic needs (Rini and Sugiharti, 2016). Anthony Hall and James Midgley (2004) convey the same thing, namely “Conditions of material and social deprivation where people fall below the minimum socially acceptable standard of living or where they experience deprivation relative to other people in society” (Yeates, 2005). The material and social deprivation that causes the individual to live below a decent standard of living or conditions in which individuals experience relative poverty compared to other individuals in society.

Poverty is conceptually divided into absolute poverty and relative poverty. Absolute poverty refers to a condition where the basic needs of a decent life are not met, both food and non-food, while relative poverty refers to the position of individuals related to the average state income (FAO, 2021). Poverty is a common problem that must be taken seriously between the government, the private sector, and the community. It is hoped that other people's concerns and awareness can help reduce poverty, so it is necessary to involve all stakeholders in poverty alleviation efforts, starting from planning, implementation, and evaluation, which are carried out on an ongoing basis (Stroe and Lincaru, 2022).

### Social entrepreneurship: From discovery to exploration

In recent years, social entrepreneurship has grown in popularity (Talić and Ivanović Dukić, 2021). Apart from Indonesia, the movement has spread to other countries, such as South Africa and South Africa. To understand social entrepreneurship, we need to define it as a concept that enables the creation of alternative business models that are market-oriented and provide social good (Terziev et al., 2020). It is crucial to realize that social entrepreneurship and entrepreneurship are fundamentally different.

There is no doubt that the social aspect of a business is more challenging to be accepted in the private sector because “social” is the opposite of “profit”. The private sector is characterized by profit-making. The concept of social entrepreneurship also contains elements of entrepreneurship. Commercial prospective entrepreneurial behavior theories understand social entrepreneurship as a multidimensional phenomenon in which social entrepreneurs, like non-profit organizations, display innovative behaviors, proactive actions, and risk management characteristics. However, the main focus lies on the company's social mission (Dwivedi and Weerawardena, 2018).

Based on the characteristics of the private sector and the emphasis on social aspects placed on social entrepreneurship, the public sector should be more responsive to social entrepreneurship. Social entrepreneurship can be applied to the public sector because the government must improve governance performance. Practicing social entrepreneurship contributes to social transformation (Cavalcanti, 2021). In Ukraine, for example, social entrepreneurship has been used by the state in developing its rural development strategy (Pechenuik, 2021). Entrepreneurial behavior in African countries continues to be poorly studied, leading to inappropriate policy actions and inadequate support (Urban, 2020).

### The stakeholder engagement and its relationship

The above understanding shows that entrepreneurs have higher self-interest and lower social awareness. Meanwhile, social entrepreneurship has lower self-interest and higher social awareness. The social entrepreneurship program involves many stakeholders. Social purpose organizations pursue multiple missions and address heterogeneous stakeholders (Siebold, 2021). Therefore, it is important for social entrepreneurs to network and communicate effectively with stakeholders. Stakeholder participation in social entrepreneurship programs is very important because it has a direct impact on organizational management (Meyer et al., 2020). An important role as a stakeholder is to achieve organizational commitment (Rodríguez-Fernández et al., 2021). Through coordination between stakeholders and communication facilitation, solutions to social problems can be resolved which lead to systemic changes (Zhao, 2020).

Stakeholder mapping can visualize stakeholder perceptions of their values and compare them with an ideal map based on social entrepreneurial missions (Burga and Rezania, 2016). Stakeholder behavior toward social entrepreneurship will influence product, service, and program innovation (Newth, 2016). In Poland, it is seen that the involvement of stakeholders from the institutional environment is crucial in promoting social entrepreneurship programs, including both formal and informal institutions (Pacut, 2020). Thus, stakeholder participation in social entrepreneurship programs is essential (Smith and Woods, 2015).

Stakeholders who have significant influence include governments, institutions, and foreign investors (Zaid et al., 2020). Institutional interactions occur in four ways: complementary, substitutive, accommodative, and competitive (Helmke and Levitsky, 2012). In this article which emphasizes the interaction of formal and informal institutions, we modify the concept of institutional interaction by applying it to the interactions between state and non-state actors.

## Methodology

The research objective is to explore the various actors involved in implementing social entrepreneurship programs in Indonesia. The various stakeholder actors studied were divided into two groups: those from government and non-governmental organizations. Our research seeks to investigate the relationship between government and non-government actors in negotiating the form of the program to be implemented. Furthermore, our research examines whether stakeholder engagement has contributed to community empowerment. This paper reports the findings of semi-structured qualitative interviews conducted with 26 informants representing all stakeholders involved in the social entrepreneurship program. Informants from elements of state institutions consist of; Ministry of Social Affairs (one person), Ministry of Industry (one person), Social Service (four people from four locations) Regional Planning Agency (four people from four locations). Meanwhile, non-state elements consist of; Social Facilitators (four people from four locations), Business Incubators (four people from four locations), Beneficiaries (four people from four locations), and Local Entrepreneurs (four people from four locations).

The interview process takes place between September and December 2022. Interviews range from 60 to 90 min, covering topics such as the role of the interviewee. At the national level, the Ministry of Social Affairs discussed the description of the social entrepreneurship program, and the Ministry of Industry discussed the role of granting business licenses. At the local government level, the Social Service discusses the implementation of the Social Entrepreneurship Program in the regions, and the Business Incubator discusses the implementation of business assistance. The social facilitator discusses social assistance for beneficiaries of the social entrepreneurship program. After that, the beneficiaries talk about their business trips.

The data collection process includes interviews (Prentice, 2017), observation (Greatorex, 2014), and documentation studies (Jones and McCoy, 2019). Interviews were conducted to gather information regarding the following topics: (a) implementation of social entrepreneurship, (b) the role of stakeholders (c) coordination between stakeholders. Documentation studies are carried out by studying reports, books, scientific journals, and other documents. This study collects data using thematic analysis (Sundler et al., 2019), presenting the collected data according to a predetermined theme, specifically stakeholder engagement. Therefore, all data collected will be aligned with stakeholder involvement in implementing social entrepreneurship.

This research has several limitations, including being conducted in four locations: Karawang Regency, Sleman Regency, Brebes Regency, and Mojokerto Regency. Thus, the situation described above does not fully represent the situation in Indonesia as a whole.

## Results

### Overview of program implementation

The Indonesian Ministry of Social Affairs has eradicated poverty through social entrepreneurship programs. In 2021, the government will implement a program to improve the poor's economic welfare. Through this program, the government helps low-income groups of people who receive conditional cash assistance programs. This program can improve the economy of low-income families and reduce their dependence on government assistance. In addition, social entrepreneurship can positively influence community members by increasing access to markets for the poor. A study of the characteristics of social enterprises has been carried out in the East African region, resulting in positive changes in marginalized groups' socio-economic and political conditions (Maseno and Wanyoike, 2020) and triggering social changes in society.

Social entrepreneurship has the potential to achieve social impact across multiple fronts, support Sustainable Development Goals, and rebalance “economic” and “social” fields (Warnecke, 2018). A study conducted in the Indian region explored the impact of social entrepreneurship on poverty alleviation (Shepherd et al., 2021). By applying a similar logic, social entrepreneurship is expected to accelerate poverty alleviation in Indonesia. This is a national development priority to reduce the burden on the poor and increase their income, especially in the bottom 40% of the population (Bappenas, 2017).

### Stakeholders involved

**Figure 1:**
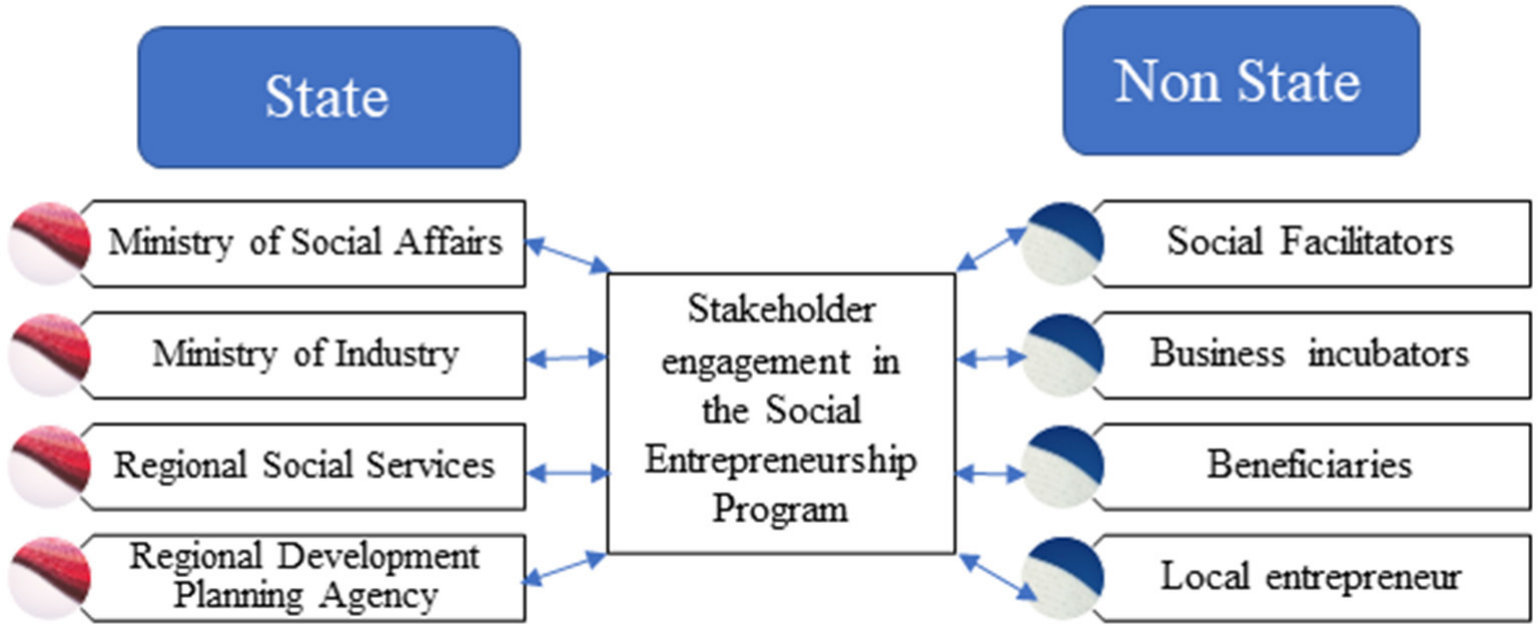
Stakeholder groups in the social entrepreneurship program, namely state and non-state.

Social entrepreneurship programs will not succeed without stakeholder involvement. The stakeholders are divided into two groups: state and non-state. Stakeholders in the social entrepreneurship program can be seen in Figure 1.

#### State actors

State actors can be grouped into two, namely, the central government and local government. This program is designed to reduce dependence on cash transfers among the poor who have received conditional cash transfers. Program beneficiaries receive capital assistance as part of the social entrepreneurship program. State elements involved in the social entrepreneurship program include the Ministry of Social Affairs, the Ministry of Industry, the Regional Development Planning Agency, and the Regional Social Service.

The Ministry of Social Affairs is a leading sector in poverty alleviation through social entrepreneurship. The Ministry of Industry plays a role in fostering production and granting business licenses. The Regional Development Planning Agency's role is to include and budget for regional development. The Regional Social Service plays a role in implementing programs in the regions. This role will continue to develop according to conditions in the area.

Countries involved in social entrepreneurship are not only concerned with economic gains but also with social goals. So that through social entrepreneurship can solve social problems (Abdullahi et al., 2020), create solutions (Kickul et al., 2014), and solve problems (Chalmers, 2021). Thus, social enterprise is relevant to social work because it aims to solve social issues (Gray et al., 2003). In this paper, the problem that is translated through social entrepreneurship is poverty.

#### Non-state actors

The Ministry of Social Affairs appointed a non-governmental organization and a university as the business incubator for the Social Entrepreneurship Program. Institutions such as these have extensive experience in assisting small and medium enterprises. Apart from the business incubator, the non-state actors involved are beneficiaries. This program is intended to help beneficiaries achieve business success.

Complementary to this model is the idea that non-state actors' existence will help solve problems that are ignored by state actors, enhancing government policy programs' effectiveness in achieving the desired outcome. As with the complementary relationship, the substitute relationship involves non-state actors' participation in achieving goals that comply with the stipulations. This is evident when state institutions cannot accomplish their objectives (Yuda et al., 2021).

In the accommodative model, non-state actors create behaviors or norms that significantly alter formal rules so that the effects are “not follow the expected results”. The latter is a competing model, which is deemed to be the most problematic. In this model, non-state actors in the program move in a direction contrary to the objectives of government policies, resulting in adverse effects. To explain how state and non-state actors interact and shape social entrepreneurship programs, we will elaborate on these four points together.

### Relations between state actors and non-state actors

Government actors and non-government actors have a dynamic relationship. It is not uncommon for them to be interdependent when non-governmental actors need government funding, while governments depend on social organizations to assist in the implementation process. Cooperation agreements between governments and non-state actors allow governments to provide services to non-state actors. By working with non-state actors, the state empowers the poor through social entrepreneurship programs involving non-state actors as program implementers. The government determines the program, in this case, the Ministry of Social Affairs, but before deciding on the program, it is important to consider the ideas of non-state stakeholders.

Initially, state and non-state actors had a hostile relationship because of their different visions and missions. Non-state actors are less satisfied because they have to comply with the rules imposed by the state. Even though at every opportunity, the state always accommodates the ideas of non-state actors. Therefore, state actors gradually began to change in response to input from non-state actors. One example of non-state dissatisfaction is the implementation of programs that have been delayed due to the COVID-19 pandemic so that the implementation time has been reduced and the targets set have been changed.

Apart from the accommodative nature of the relationship, there is also a tendency for a substitutionary relationship because non-state actors are more experienced in empowering communities through social entrepreneurship programs with limited scope. The state has the power to finance in a complete sense. Thus, the relationship between planning and funding is accommodative. However, technically the implementation of the program is more of a substitute.

### Social entrepreneurship effects resulting from stakeholder engagement

The policies taken by the government to limit crowds during the COVID-19 pandemic certainly had an impact on MSMEs, including social entrepreneurs. It was noted that turnover decreased following the implementation of the lockdown and working-from-home policies. There has been a decrease in the number of business buyers run by beneficiaries because many fear contracting the Coronavirus. Food usually bought and consumed outside has been diverted to be cooked at home by consumers concerned about the quality of their food.

According to previous research from the Social Welfare Center, there are three conditions for receiving benefits: profits before the pandemic, benefits during the pandemic before joining the program, and benefits after participating in the program during a pandemic. Before the pandemic, the average profit was between IDR 50,000 and 99,000, the study found. During the pandemic, the average profit fell below IDR 50,000. They increased their earnings to between IDR 50,000 and 99,000 through the Social Entrepreneurship Program. Because of this, the new social entrepreneurship program has proven capable of returning to pre-pandemic conditions (Setiawan et al., 2021).

## Discussion

Social entrepreneurship programs cannot run effectively without stakeholder involvement. The state's involvement is the driving force for all stakeholders in planning, budgeting, mentoring, and monitoring. Social entrepreneurship incubators originating from non-state actors are executors of technical programs from state programs. Initially, the social entrepreneurship program initiated by the central government was rejected by regional stakeholders because it was considered competition. However, through an informal discussion process, each stakeholder realized that there were similarities that became strengths during the discussion process. This meeting point is so that social entrepreneurship programs from anywhere, including from the central government, can be included in the program scheme designed by the regional government and even be developed further in the future.

Furthermore, we found that the government's concept of empowerment still mixes well with the idea of social assistance. Beneficiaries should receive social assistance in an emergency or if they are in a position where they cannot survive on their own. If these conditions are not met, empowerment programs will not be effective since they are concerned with fulfilling their basic needs. Empowerment programs are most appropriate for those whose basic needs have already been met, even if they are being met by social assistance. When persons whose basic needs have not been met are provided with empowerment, they tend to have little motivation to participate in a program. There is an alternative strategy to encourage friendship networks to internalize social risk since the replacement of welfare regimes from products has not been completed (Yuda, 2018, 2021; Yuda et al., 2021). These arrangements reflect the prevailing welfare regime in the Global South, where informal relationships interact with statutory provisions and are often conditioned to provide social welfare.

The Social Entrepreneurship Program combines two concepts in the Ministry of Social Affairs: empowerment and social protection. Capital assistance is considered to be a type of social protection program. Some evidence suggests that beneficiaries are motivated to participate in the program due to the service provided. However, social entrepreneurship programs can lead to dependency on capital assistance. A key component of strengthening the social entrepreneurship program without increasing dependence is contributing substantially to the beneficiaries. The mentoring beneficiaries will receive guidance in running their businesses through good planning.

## Conclusion

In Indonesia, social entrepreneurship programs tend to be driven by the state because the paradigm of social entrepreneurship has not been widespread among the private sector or other non-state actors. In contrast, in developed countries view social entrepreneurship as a moral obligation. It is important to note that social entrepreneurship programs in developing countries are not just empowerment programs, even though they involve stakeholders, because capital assistance is still provided. These programs may have arisen as a result of beneficiary dependence on aid.

Beneficiaries who participate in programs launched by the government may receive services not because they are struggling to get out of poverty. Empowerment is defined as a strategy through effective planning so that beneficiaries do not depend on government assistance. Scientifically, the transformation from social assistance to empowerment is not easy to do. It takes a long time in the empowerment process so that beneficiaries can be empowered. this is the limitation of this research because the program is implemented in one fiscal year, so the impact of the program is not yet clearly visible. Interesting future research to study is the meaning of beneficiaries of the empowerment program. If the meaning is still like social assistance then the purpose of empowerment will not be achieved.

## Nomenclature

-Resource Identification Initiative

-Life Science Identifiers

## Author contributions

All authors listed have made a substantial, direct, and intellectual contribution to the work and approved it for publication.

## References

[B1] Abdullahi H. M. Sambo H. Abubakar A. A. (2020). Social entrepreneurship as a means of eradicating social problems in gombe metropolis. 2507, 1–9.

[B2] Abrar ul haq M. Jali M. R. M. Islam G. M. N. (2019). Household empowerment as the key to eradicate poverty incidence. Asian Soc. Work Policy Rev. 13, 4–24. 10.1111/aswp.1215235095171

[B3] Bappenas. (2017). Prakarsa pemerintah daerah dalam upaya pengurangan kesenjangan wilayah dan pembangunan daerah (Issue 2).

[B4] Bradshaw T. K. (2009). Theories of poverty and anti-poverty programs in community development. Commun. Develop. 38, 7–25. 10.1080/1557533070949018231874751

[B5] Burga R. Rezania D. (2016). Stakeholder theory in social entrepreneurship: a descriptive case study. J. Global Entrepreneur. Res. 6. 10.1186/s40497-016-0049-8

[B6] Cavalcanti M. F. R. (2021). Social entrepreneurship and social change: a practice-based study in non-governmental organizations. RAUSP Manag. J. 56, 170–185. 10.1108/RAUSP-05-2020-0091

[B7] Chalmers D. (2021). Social entrepreneurship's solutionism problem. J. Manag. Stud. 58, 1363–1370. 10.1111/joms.12676

[B8] Doh S. (2020). Social entrepreneurship and regional economic development: the case of social enterprise in south korea. Sustainability 12, 1–20. 10.3390/su1221884335136666

[B9] Dwivedi A. Weerawardena J. (2018). Conceptualizing and operationalizing the social entrepreneurship construct. J. Bus. Res. 86, 32–40. 10.1016/j.jbusres.2018.01.053

[B10] FAO (2021). Ending Poverty and Hunger by the Context Strategic Investments for Achieving SDG 1 and SDG 2 Examples of Strategic Investments For Poverty Reduction. Available online at: https://www.fao.org/documents/card/en/c/29670e1a-f025-49cf-bfaf-82fef4baa1b9/ (accessed February 01, 2023).

[B11] Govender P. Kambaran N. Patchett N. Ruddle A. Torr G. Zyl N. van. (2007). Poverty and inequality in South Africa and the world. South African Actuarial J. 7, 117–160. 10.4314/saaj.v7i1.24511

[B12] Gray M. Healy K. Crofts P. (2003). Social enterprise: is it the business of social work? Aust. Soc. Work 56, 141–154. 10.1046/j.0312-407X.2003.00060.x

[B13] Greatorex M. (2014). “Observation,” in Wiley Encyclopedia of Management, ed C. L Cooper (John Wiley & Sons, Ltd).

[B14] Helmke G. Levitsky S. (2012). Informal institutions and comparative politics: a research agenda. Int. Handbook Inf. Govern. 2, 85–113. 10.4337/9781781001219.0001128388621

[B15] Jones K. M. L. McCoy C. (2019). Reconsidering data in learning analytics: opportunities for critical research using a documentation studies framework. Learn. Media Technol. 44, 52–63. 10.1080/17439884.2018.1556216

[B16] Kickul J. R. Griffiths M. D. Lisa K. (2014). social entrepreneurship. Wiley Encycl. Manag. 10.1002/9781118785317.weom030087

[B17] Laborde D. Martin W. Vos R. (2021). Impacts of COVID-19 on global poverty, food security, and diets: Insights from global model scenario analysis. Agri. Econom. (Amsterdam, Netherlands). 52, 375–390. 10.1111/AGEC.12624 34230728 PMC8251321

[B18] Lateh M. Hussain M. D. Abdullah M. S. (2018). Social entrepreneurship development and poverty alleviation - a literature review. J. Bus. Manag. 2, 1–11.

[B19] Maseno M. Wanyoike C. (2020). Social entrepreneurship as mechanisms for social transformation and social impact in east africa an exploratory case study perspective. J. Soc. Entrep. 1–26. 10.1080/19420676.2020.1755348

[B20] Meyer C. R. Cohen D. G. Gauthier J. (2020). Social entrepreneurship, stakeholder management, and the multiple fitness elements of sustainability: where cash is no longer king. J. Small Bus. Entrepreneur. 32, 431–455. 10.1080/08276331.2019.1661614

[B21] Mustaffa C. S. Halim H. Ahmad J. Ishak Q. Johari N. A. (2020). Disability and poverty: a review on social entrepreneurship opportunities for persons with disabilities in Malaysia. Albukhary Soc. Bus. J. 1, 1–11. 10.55862/asbjV1I2a001

[B22] Newth J. (2016). Social enterprise innovation in context: stakeholder influence through contestation. Entrepreneur. Res. J. 6, 369–399. 10.1515/erj-2014-0029

[B23] Ngare I. (2021). Green innovation enterprises and environmental entrepreneurship for poverty alleviation in Kenya. Preprints 2021, 2021050469. 10.20944/preprints202105.0469.v132283112

[B24] Pacut A. (2020). Drivers toward social entrepreneurs engagement in poland: an institutional approach. Administr. Sci. 10, 5. 10.3390/admsci10010005

[B25] Pechenuik A. (2021). Social entrepreneurship as a factor in the development of rural communities in Ukraine. VUZF Rev. 6, 111–118. 10.38188/2534-9228.21.2.13

[B26] Prentice C. M. (2017). “Interviews, qualitative,” in The International Encyclopedia of Intercultural Communication. p. 1–8. 10.1002/9781118783665.ieicc0108

[B27] Rini A. S. Sugiharti L. (2016). Determining factors of poverty in Indonesia: Household analysis. JIET (Jurnal Ilmu Ekonomi Terapan). 1, 80–95. 10.20473/JIET.V1I2.3252

[B28] Rodríguez-Fernández M. Gaspar-González A. I. Sánchez-Teba E. M. (2021). Sustainable social responsibility through stakeholders engagement. Corp. Soc. Respons. Environ. Manag. 10.1002/csr.2023

[B29] Sadabadi A. A. Rahimi Rad Z. (2021). Social innovation participatory action research for empowerment of marginalized people. Asian Soc. Work Policy Rev. 15, 160–172. 10.1111/aswp.12228

[B30] Setiawan H. H. Nuryana M. Susantyo B. Purwanto A. B. Sulubere M. B. (2021). Social entrepreneurship for beneficiaries of the Program Keluarga Harapan (PKH) toward sustainable development. IOP Conf. Ser. Earth Environ. Sci. 739, 012053. 10.1088/1755-1315/739/1/012053

[B31] Shepherd D. A. Parida V. Wincent J. (2021). Entrepreneurship and poverty alleviation: The importance of health and children's education for slum entrepreneurs. Entrepreneursh.: Theor. Pract. 45, 350–385. 10.1177/1042258719900774

[B32] Siebold N. (2021). Reference points for business model innovation in social purpose organizations: a stakeholder perspective. J. Bus. Res. 125, 710–719. 10.1016/j.jbusres.2020.01.032 PMC7456298 32904448

[B33] Smith L. Woods C. (2015). Stakeholder engagement in the social entrepreneurship process: identity, governance and legitimacy. J. Soc. Entrepreneur. 6, 186–217. 10.1080/19420676.2014.987802

[B34] Stroe C. Lincaru C. (2022). Brief feature of poverty and rural poverty and the circle of decline in romanian rural area. J. Econ. Soc. Dev. 9, 25–35. 10.55539/JESD.9.1.4

[B35] Sundler A. J. Lindberg E. Nilsson C. Palmér L. (2019). Qualitative thematic analysis based on descriptive phenomenology. Nurs Open 6, 733–739. 10.1002/nop2.275 31367394 PMC6650661

[B36] Talić M. Ivanović Dukić M. (2021). Comparative analysis of developmental concepts of social entrepreneurship in Europe and the USA. Facta Univ. Ser. 17, 385. 10.22190/FUEO200907028T

[B37] Terziev V. Bencheva N. Stoeva T. Georgiev M. (2020). Developing social entrepreneurship in the EU: a cross-country analysis. SSRN Electron. J. VI, 37–44. 10.2139/ssrn.3525681

[B38] Urban B. (2020). Entrepreneurial alertness, self-efficacy and social entrepreneurship intentions. J. Small Bus. Enterprise Dev. 27, 489–507. 10.1108/JSBED-08-2019-0285

[B39] Warnecke T. (2018). Social entrepreneurship in China: driving institutional change. J. Econ. Issues 52, 368–377. 10.1080/00213624.2018.146986635545782

[B40] Yeates N. (2005). Anthony Hall and James Midgley (2004), Social Policy for Development, London: Sage, 288 pp., 322.99 pbk, ISBN 0-7619-6715-X. J. Soc. Policy 34, 312–313. 10.1017/S0047279405228805

[B41] Yuda T. K. (2018). Welfare regime and the patrimonial state in contemporary Asia: visiting Indonesian cases. J. Asian Public Policy 12, 351–365. 10.1080/17516234.2018.1462685

[B42] Yuda T. K. (2021). The complementary roles between clientelism and familism in social policy development. Soc. Policy Administr. 55, 1370–1392. 10.1111/spol.12738

[B43] Yuda T. K. Pratiyudha P. P. Kafaa K. A. (2021). Managing social policy in the emerging welfare regime of governance: what Indonesia can learn from South Korea's experience. Int Soc. Work. 10.1177/00208728211011634

[B44] Zaid M. A. A. Abuhijleh S. T. F. Pucheta-Martínez M. C. (2020). Ownership structure, stakeholder engagement, and corporate social responsibility policies: the moderating effect of board independence. Corp. Soc. Respons. Environ. Manag. 27, 1344–1360. 10.1002/csr.1888

[B45] Zhao M. (2020). Social entrepreneurship for systemic change: the case of Southeast and South Asian countries. J. Asian Public Policy 14, 1–25. 10.1080/17516234.2020.1796325

